# HIV seroprevalence among participants at a Supervised Injection Facility in Vancouver, Canada: implications for prevention, care and treatment

**DOI:** 10.1186/1477-7517-3-36

**Published:** 2006-12-18

**Authors:** Mark W Tyndall, Evan Wood, Ruth Zhang, Calvin Lai, Julio SG Montaner, Thomas Kerr

**Affiliations:** 1 Department of Medicine, University of British Columbia, Vancouver Hospital, 2775 Laurel Street, Vancouver, V5Z 1M9, Canada; 2 BC Centre for Excellence in HIV/AIDS, St. Paul's Hospital, 1081 Burrard Street, Vancouver, V6Y 1Y6, Canada

## Abstract

North America's first government sanctioned medically supervised injection facility (SIF) was opened during September 2003 in Vancouver, Canada. This was in response to a large open public drug scene, high rates of HIV and hepatitis C transmission, fatal drug overdoses, and poor health outcomes among the city's injection drug users. Between December 2003 and April 2005, a representative sample of 1,035 SIF participants were enrolled in a prospective cohort that required completing an interviewer-administered questionnaire and providing a blood sample for HIV testing. HIV infection was detected in 170/1007 (17%) participants and was associated with Aboriginal ethnicity (adjusted Odds Ratio [aOR], 2.70, 95% Confidence Interval [95% CI], 1.84–3.97), a history of borrowing used needles/syringes (aOR, 2.0, 95% CI, 1.37–2.93), previous incarceration (aOR, 1.87, 95% CI, 1.11–3.14), and daily injection cocaine use (aOR, 1.42, 95% CI, 1.00–2.03). The SIF has attracted a large number of marginalized injection drug users and presents an excellent opportunity to enhance HIV prevention through education, the provision of sterile injecting equipment, and a supervised environment to self-inject. In addition, the SIF is an important point of contact for HIV positive individuals who may not be participating in HIV care and treatment.

## Background

In response to a large open public drug scene, high rates of HIV and hepatitis C transmission, fatal drug overdoses, and poor health outcomes among injection drug users, Vancouver established North America's first government sanctioned medically supervised safer injection facility (SIF) in September 2003 [1-3]. The SIF has been approved as a three year scientific evaluation by Health Canada with a predetermined set of outcomes to be evaluated through a comprehensive prospective strategy [4,5]. Initial findings from the evaluation have been published, including evidence that the SIF has attracted a wide range of marginalized injection drug users (IDUs) [6,7], has reduced drug related public disorder [8], and has been associated with reduced syringe sharing [9,10].

With respect to HIV, the focus of the SIF to date, as with other harm reduction initiatives, has been on reducing HIV transmission through the provision of sterile syringes and providing a space where self-administered injections can be conducted in a clean and controlled environment [4,11]. It has been previously shown in this community that HIV infection has a disproportional impact on injection cocaine users [12], women [13], and those of Aboriginal ethnicity [14], and efforts to specifically engage and accommodate these groups at the SIF are ongoing. Given the high representation of these groups at the SIF, it is anticipated that attending the SIF will result in reduced transmission of HIV.

The purpose of this analysis is to measure the prevalence and correlates of baseline HIV among those who are using the SIF. This information is important to determine if the SIF could be used as a site for HIV related care and treatment. This is also important in order to measure the longitudinal incidence of HIV transmission among those using the SIF.

## Methods

As part of a comprehensive evaluation strategy, a representative cohort of SIF users (SEOSI) was recruited and followed prospectively. The methods have been described previously [5]. Briefly, the cohort includes SIF users who were selected through a random number generation strategy. Each week between 16 and 32 two-hour time blocks were designated for recruitment between the opening hours of 10:00 a.m. and 4:00 a.m. seven days per week. During these random time periods 10 cards were distributed to consecutive SIF users who were invited to visit the SEOSI cohort study office located one block from the SIF. There was a CAN$20 compensation provided if they were willing to participate in the prospective study following a full explanation, providing a written informed consent, completing an interviewer-administered questionnaire and supplying a blood sample for HIV and hepatitis C testing. All SEOSI participants provide informed consent to link to the Insite database so that SIF use can be tracked, as well as informed consent to access administrative health record databases in the community. The study was closed to new participants as of March 31, 2005 at which time 1,035 people were enrolled in the cohort from 4,764 individuals who had ever visited the SIF. A comparison between all SIF users and SEOSI cohort participants has shown statistically similar socio-demographic variables (all *p *> 0.5)[5]. The study was approved by the University of British Columbia/Providence Health Care Ethics Board.

To determine factors associated with HIV infection, bivariate analysis was performed using Pearson's Chi-square testing and Wilcoxon rank sum test. Logistic regression analysis was also performed to examine factors that were independently associated with HIV infection. The multivariable models were fit adjusting for variables that were of interest *a priori *or that were statistically significant at the p < 0.05 level in the bivariable analyses. The statistical analysis was performed using SPSS 12.0, and all reported p-values are two sided.

## Results

This analysis includes data from the baseline recruitment of 1,035 individuals who were randomly selected to participate in the SEOSI cohort between December 1, 2003 and March 31, 2005. Of these, HIV testing was available on 1007 (97%). The missing HIV results were attributed to difficulty in obtaining venous blood samples from 28 of the participants. Among those tested, 170 of 1007 (17%) were found to be HIV positive. Table 1 shows the demographic characteristics of the participants stratified by HIV serostatus. In this bivariate comparison, HIV positive status was associated with more years of drug injecting (p = 0.008), Aboriginal ethnicity (p < 0.001), daily cocaine injecting (p = 0.020), borrowing used needles/syringes (p < 0.001), methadone maintenance treatment (p = 0.018), sex work (p = 0.051), and history of incarceration (p = 0.004). In this cohort, HIV infection was not associated with gender, residence in the Vancouver's Downtown Eastside, daily heroin injection, daily crystal methamphetamine injection, public drug use, requiring help with injecting, sharing other drug using equipment, or binge drug use.

**Table 1:** Prevalence of HIV stratified by socio-demographic and behavioural variables.

**Characteristic**	**HIV-Positive n (%)**	**HIV-Negative n (%)**	**Odds Ratio (95% CI)**	***p *value**
**Age**				
Median (IQR)	37.9 (10.3)	38.6 (12.1)		.914
**Gender**				
Male	113 (66.5)	612 (73.1)	0.73 (0.51 – 1.04)	.078
Female	57 (33.5)	225 (26.9)		
**Ethnicity**				
Aboriginal	55 (32.4)	140 (16.7)	2.38 (1.65 – 3.44)	<.001
Other	115 (67.6)	697 (83.3)		
**Reside in DTES**				
Yes	120 (70.6)	570 (68.1)	1.12 (0.78 – 1.61)	.524
No	50 (29.4)	267 (31.9)		
**Daily Cocaine Injection**				
Yes	68 (40.0)	258 (30.8)	1.50 (1.07 – 2.10)	.020
No	102 (60.0)	579 (69.2)		
**Daily Heroin Injection**				
Yes	78 (45.9)	435 (52.0)	0.78 (0.56 – 1.09)	.148
No	92 (54.1)	402 (48.0)		
**Daily Crystal Meth Injection**				
Yes	3 (1.8)	31 (3.7)	0.47 (0.14 – 1.55)	.202
No	167 (98.2)	806 (96.3)		
**Public drug use**				
Yes	128 (75.3)	605 (72.3)	1.17 (0.80 – 1.71)	.421
No	42 (24.7)	232 (27.7)		
**Ever borrow needles/syringes**				
Yes	122 (71.8)	455 (54.4)	2.13 (1.49 – 3.06)	<.001
No	48 (28.2)	382 (45.6)		
**Share other equipment**				
Yes	104 (61.2)	477 (57.0)	1.19 (0.85 – 1.67)	.314
No	66 (38.8)	360 (43.0)		
**Require help injecting**				
Yes	134 (78.8)	619 (74.0)	1.31 (0.88 – 1.95)	.183
No	36 (21.2)	218 (26.0)		
**Binge drug use**				
Yes	109 (64.1)	525 (62.7)	1.06 (0.75 – 1.50)	.732
No	61 (35.9)	312 (37.3)		
**Addiction Treatment**				
Yes	92 (54.1)	361 (43.1)	1.56 (1.12 – 2.17)	.009
No	78 (45.9)	476 (56.9)		
**On Methadone Currently**				
Yes	48 (28.2)	168 (20.1)	1.57 (1.08 – 2.28)	.018
No	122 (71.8)	669 (79.9)		
**Sex-trade Ever**				
Yes	78 (45.9)	317 (37.9)	1.39 (1.00 – 1.94)	.051
No	92 (54.1)	520 (62.1)		
**History of incarceration**				
Yes	150 (88.2)	658 (78.6)	2.04 (1.24 – 3.35)	.004
No	20 (11.8)	179 (21.4)		

Note: IQR = inter-quartile range, DTES = Downtown Eastside

In the logistic regression analysis shown in Table 2, HIV positive status was independently associated with Aboriginal ethnicity (adjusted Odds Ratio [aOR] 2.70, 95% Confidence Interval [CI] 1.84, 3.97), borrowing used needles/syringes (aOR = 2.00, 95% CI:1.37, 2.93), history of incarceration (aOR = 1.87, 95% CI:1.11, 3.14), and daily cocaine injection (aOR 1.42, 95% CI:1.00, 2.03).

**Table 2:** Multivariate Logistic Regression Analysis of Factors associated with baseline HIV Infection.

**Characteristic**	**Adjusted Odds Ratio**	**95% C.I.**	***p*-value**
**Aboriginal ethnicity**			
Yes vs No	2.70	1.84 – 3.97	<.001
**Ever borrow needles/syringes**			
Yes vs No	2.00	1.37 – 2.93	<.001
**History of incarceration**			
Yes vs No	1.87	1.11 – 3.14	.019
**Daily Cocaine Use**			
Yes vs No	1.42	1.00 – 2.03	.050

## Discussion

The overall HIV seroprevalence among a random cohort of injection drug users attending the SIF was 17%. This was not unexpected as high rates of HIV infection among injection drug users has been reported in this community for over a decade [1,12]. However, the random selection process used to assemble this cohort may be more representative of active injection drug users in this community when compared with previous estimates that were based on non-random recruitment. The variables associated with HIV infection in this cohort; Aboriginal ethnicity, borrowing used needles, incarceration, and cocaine use, are consistent with characteristics previously described in this population.

The disproportionately high HIV prevalence among Aboriginal people has been attributed to the convergence of environmental, social and behavioral factors that increase vulnerability to illicit drug use and HIV infection [14,15]. Providing culturally relevant services for Aboriginal people is a priority for this community as the uptake of services and supports is suboptimal. In this context, it is encouraging that the SIF has attracted a relatively large number of Aboriginal people, and can provide an important point of contact for those who may be reluctant to participate in other health and social services.

The association between intensive cocaine use and HIV infection has been well described in this community and injection cocaine is consistently found to increase HIV transmission [12,16]. The propensity of many IDUs to use cocaine in high-intensity episodic patterns contributes to the high risk of HIV transmission associated with cocaine use [17]. This pattern of drug use may be particularly influenced at the SIF as only one injection is allowed at each visit. This may pre-empt a prolonged "drug-run" or individuals may decide to use the SIF specifically as a way to interrupt a current period of intensive drug use. Studies are currently underway to better understand the impact on the SIF on drug use patterns. These results however do show that cocaine users do attend the SIF and that earlier concerns that people would not use cocaine at the SIF were unfounded [6].

A history of incarceration is often an indicator of social isolation and the majority of convictions seen in this population are on the basis of illegal drug infractions. The relationship between incarceration and increased HIV transmission among injection drug users is a major area of debate for Canada and globally [18]. In this cross-sectional study, it is not possible to determine the date of HIV infection and its temporal relationship with prior incarceration, however there are risk behaviors that do occur during the time of incarceration and more efforts to reduce the harms to inmates are needed [19-21].

In addition to connecting with HIV positive people, the SIF functions as an important entry point to provide primary HIV prevention. One of the primary objectives of the SIF is to develop consistent contact with people at risk of HIV who are often isolated and marginalized. The SIF offers an engaging, low threshold environment and participants are encouraged to attend regularly. During the visits there is an opportunity to offer HIV prevention education through the use of sterile injection techniques and to emphasize the importance of clean needles as well as opportunities for referral to addiction services including counseling, detoxification, and methadone programs [6].

It would be extremely unlikely to be exposed to HIV while injecting at the SIF. All participants are supplied with new needles/syringes, alcohol swabs, elastic tourniquets, and cookers if required. All injections occurring within the SIF are restricted to self-injections and this eliminates the high risk behavior of people injecting each other [22]. However, this restriction will deter those who do require help injecting from attending the SIF and strategies to reach this group of IDUs are needed. Despite the high attendance at the SIF, for many participants the majority of injections occur in other locations that may lead to risky drug use practices. The site is currently operating at capacity with approximately 700 visits per day. Increased hours of operation (i.e. from 18 to 24 hours per day) and greater capacity to accommodate more injection drug users within the SIF would increase coverage.

There are a number of limitations with this study. The cross-sectional nature of the analysis does not allow the timing of HIV transmission to be determined and thus some of the associated risks may have occurred after the HIV infection. Secondly, some of the risk variables were based on self-report and this may have been biased by socially desirable responses. Thirdly, the participants in the study were selected from those who had already made a decision to use the SIF and are not necessarily representative of the injection drug using community.

Our results demonstrate a 17% prevalence of HIV infection among a representative cohort of IDUs who attend Vancouver's SIF. The SIF has successfully attracted a group of marginalized HIV infected individuals and therefore provides a unique opportunity to improve access to health services and HIV care and treatment [23]. Furthermore, the capacity to prevent new cases of HIV through enhanced prevention messages and interventions at the SIF has great potential. Many cities are confronting the serious health and social consequences of poorly controlled injection drug use among marginalized citizens and subsequent outbreaks of HIV infection. The SIF in Vancouver has provided a valuable addition to existing services for injection drug users and should be considered in other communities.
